# Supplementary material for: Utility of Genetic Testing from the Perspective of Parents/Caregivers: A Scoping Review
Source: Children (Basel). 2021 Mar 27;8(4):259. doi: 10.3390/children8040259 (PMC8067127; doi:10.3390/children8040259)
Supplement: Supplementary file 1 [file children-08-00259-s001.pdf]

Supplementary File:

Figure S1. Search Strategy.

The full list of search terms included: *Genetic Testing/ [OR] exome sequencing.tw,kf. [OR] genetic counseling.tw,kf. [OR] genetic counselling.tw,kf. [OR] genetic information.tw,kf. [OR] genetic predictive test$.tw,kf. [OR] genetic predisposition test$.tw,kf. [OR] genetic screen$.tw,kf. [OR] genetic sequencing.tw,kf. [OR] genetic test$.tw,kf. [OR] genome based test$.tw,kf. [OR] genomic information.tw,kf. [OR] genomic sequencing.tw,kf. [OR] genom$ test$.tw,kf. [OR] genomic? profiling.tw,kf. [OR] "direct to consumer testing".tw,kf. [OR] "direct to consumer genetic?".tw,kf. [OR] "direct to consumer genomic?".tw,kf. [AND] patient outcome assessment/ [OR] patient reported outcome measures/ [OR] Patient Preference/ [OR] caregiver utility.tw,kf. [OR] clinical meaning.tw,kf. [OR] clinical utility.tw,kf. [OR] clinical value?.tw,kf. [OR] conjoint analys#s.tw,kf. [OR] consumer meaning.tw,kf. [OR] consumer utility.tw,kf. [OR] consumer value.tw,kf. [OR] discrete choice experiment?.tw,kf. [OR] famil$ utility.tw,kf. [OR] health$ utility.tw,kf. [OR] health-related utility.tw,kf. [OR] individual meaning.tw,kf. [OR] individual utility.tw,kf. [OR] individual value.tw,kf. [OR] medical meaning.tw,kf. [OR] medical utility.tw,kf. [OR] medical value?.tw,kf. [OR] nonclinical benefit?.tw,kf. [OR] non-clinical benefit?.tw,kf. [OR] nonclinical harm?.tw,kf. [OR] non-clinical harm?.tw,kf. [OR] nonmedical benefit?.tw,kf. [OR] non-medical benefit?.tw,kf. [OR] nonmedical harm?.tw,kf. [OR] non-medical harm?.tw,kf. [OR] parent$ utility.tw,kf. [OR] parent$ useful$.tw,kf. [OR] parent$ perception?.tw,kf. [OR] parent$ experience?.tw,kf. [OR] parent$ value?.tw,kf. [OR] participant meaning.tw,kf. [OR] participant utility.tw,kf. [OR] participant$ value?.tw,kf. [OR] patient meaning?.tw,kf. [OR] patient reported outcome?.tw,kf. [OR] patient? utility.tw,kf. [OR] patient? value?.tw,kf. [OR] personal benefit?.tw,kf. [OR] personal harm?.tw,kf. [OR] personal meaning?.tw,kf. [OR] personal outcome?.tw,kf. [OR] personal utility.tw,kf. [OR] personal value?.tw,kf. [OR] public meaning.tw,kf. [OR] public utility.tw,kf. [OR] public value?.tw,kf. [OR] ("willing to pay$" [OR] "willingness to pay$").tw,kf.

Table S1. Quality appraisal scores.

**Qualitative studies**																								
**Assigned study number**	**1**	**3**	**4**	**5**	**6**	**7**	**9**	**10**	**11**	**12**	**13**	**15**	**16**	**21**	**23**	**24**	**26**	**30**	**31**	**33**	**18***	**20***	**27***	**28***
1) Question/objective sufficiently described?	2	2	2	2	1	2	2	1	2	2	1	1	2	2	2	1	2	2	2	2	2	2	2	2
2) Study design evident and appropriate?	2	2	2	2	2	2	2	2	2	2	2	2	2	2	2	1	2	2	2	2	2	2	2	2
3) Context for study clear?	2	2	2	2	2	1	1	2	1	2	2	2	2	2	2	2	2	2	2	2	2	2	2	2
4) Connection to a theoretical framework/wider body of knowledge?	2	2	1	2	1	1	2	1	1	2	2	2	2	1	2	2	2	2	2	2	1	1	2	2
5) Sampling strategy described, relevant and justified?	1	2	1	2	2	1	0	0	1	2	1	0	1	1	1	2	2	2	2	1	1	1	2	2
6) Data collection methods clearly described and systematic?	1	2	1	2	2	2	1	2	1	1	1	1	2	2	2	2	2	2	2	1	2	2	2	1
7) Data analysis clearly described and systematic?	2	2	2	2	2	2	2	2	1	2	2	2	1	2	2	2	2	2	2	2	2	1	2	1
8) Use of verification procedure(s) to establish credibility?	0	2	2	2	2	2	2	2	2	2	0	2	2	2	2	0	2	2	2	2	2	0	2	0

9) Conclusions supported by the results?	2	2	2	2	2	2	1	2	1	2	2	1	1	1	2	1	2	2	2	2	2	2	2	2
10) Reflexivity of the account?	1	1	1	1	1	1	1	0	1	1	1	0	1	1	0	0	1	1	1	1	1	1	0	1
Total	15	19	16	19	17	16	14	14	13	18	14	13	16	16	17	13	19	19	19	17	17	14	18	15
Out of	20	20	20	20	20	20	20	20	20	20	20	20	20	20	20	20	20	20	20	20	20	20	20	20
**Percent score (%)**	**75**	**95**	**80**	**95**	**85**	**80**	**70**	**70**	**65**	**90**	**70**	**65**	**80**	**80**	**85**	**65**	**95**	**95**	**95**	**85**	**85**	**70**	**90**	**75**

*Mixed methods papers were not included in the calculation of the average qualitative quality score

**Quantitative studies**													
**Assigned study number**	**2**	**8**	**14**	**17**	**19**	**22**	**25**	**29**	**32**	**18***	**20***	**27***	**28***
1) Question/objective sufficiently described?	2	2	2	2	2	2	2	2	2	1	2	2	2
2) Study design evident and appropriate?	2	2	2	2	2	2	2	2	2	2	2	2	2
3) Method of subject/comparison group selection or source of information/input variables described and appropriate?	1	2	1	1	2	1	2	2	1	2	2	2	2
4) Subject (and comparison group, if applicable) characteristics sufficiently described?	1	2	1	2	1	2	2	2	2	2	2	2	1
5) If interventional and random allocation was possible, was it described?	N/A	N/A	N/A	N/A	N/A	N/A	N/A	2	N/A	N/A	1	N/A	N/A
6) If interventional and blinding of investigators was possible, was it reported?	N/A	N/A	N/A	N/A	N/A	N/A	N/A	N/A	N/A	N/A	2	N/A	N/A
7) If interventional and blinding of subjects was possible, was it reported?	N/A	N/A	N/A	N/A	N/A	N/A	N/A	N/A	N/A	N/A	2	N/A	N/A
8) Outcome and (if applicable) exposure measure(s) well de-	1	2	2	1	2	2	2	2	2	1	1	2	2

fined and robust to measurement/misclassification bias? Means of assessment reported?													
9) Sample size appropriate?	2	2	2	0	2	2	2	1	2	1	1	1	1
10) Analytic methods described/justified and appropriate?	2	2	1	2	2	2	2	2	2	2	N/A	2	2
11) Some estimate of variance is reported for the main results?	0	2	1	2	2	2	2	2	2	2	0	2	0
12) Controlled for confounding?	1	2	N/A	2	0	0	1	1	2	2	1	1	1
13) Results reported in sufficient detail?	1	1	2	1	2	2	2	2	2	2	2	2	2
14) Conclusions supported by the results?	1	2	1	2	2	2	2	2	2	2	2	2	2
Total	14	21	15	17	19	19	21	22	21	19	20	20	17
Out of	22	22	20	22	22	22	22	24	22	22	26	22	22
**Percent score (%)**	**63.6**	**95.5**	**75.0**	**77.3**	**86.4**	**86.4**	**95.5**	**91.6**	**95.5**	**86.4**	**76.9**	**90.9**	**77.3**

*Mixed methods papers were not included in the calculation of the average quantitative quality score

**MMAT**				
**Assigned study number**	**18**	**20**	**27**	**28**
1) Is there an adequate rationale for using a mixed methods design to address the research question	0	2	2	2
2) Are the different components of the study effectively integrated to answer the research question?	1	2	2	2
3) Are the outputs of the integration of qualitative and quantitative components adequately interpreted?	1	1	2	2

4) Are divergences and inconsistencies between quantitative and qualitative results adequately addressed?	0	1	2	2
5) Do the different components of the study adhere to the quality criteria of each tradition of the methods involved?	2	2	2	1
MMAT total	4	8	8	9
MMAT out of	10	10	10	10
Qualitative total	17	14	18	15
Quantitative total	19	20	20	17
Mixed methods total (MMAT + Qual + Quant)	40	42	48	41
Out of	52	56	52	52
**Percent score (%)**	**76.9**	**75.0**	**92.3**	**78.8**

Table S2. Scoping review studies and assigned study number.

Assigned Study Number	First Author + Year	Study Title
1	Chudleigh 2016 [30]	Parents' experiences of receiving the initial positive Newborn Screening (NBS) result for Cystic Fibrosis and Sickle Cell Disease
2	Gebhardt 2016 [29]	How do patient perceived determinants influence the decision-making process to accept or decline preimplantation genetic screening?
3	Hayeems 2016 [15]	Parents' experience with pediatric microarray: Transferrable lessons in the era of genomic counseling
4	Hodgson 2016 [46]	Experiences of prenatal diagnosis and decision-making about termination of pregnancy: A qualitative study
5	Kerruish 2016 [45]	Parents' experiences 12 years after newborn screening for genetic susceptibility to type 1 diabetes and their attitudes to whole-genome sequencing in newborns
6	Krabbenborg 2016a [44]	Evaluating a counselling strategy for diagnostic WES in paediatric neurology: an exploration of parents' information and communication needs
7	Krabbenborg 2016b [43]	Understanding the psychosocial effects of WES test results on parents of children with rare diseases
8	Lingen 2016 [42]	Obtaining a genetic diagnosis in a child with disability: impact on parental quality of life
9	Rosell 2016 [41]	Not the end of the odyssey: Parental perceptions of Whole Exome Sequencing (WES) in pediatric undiagnosed disorders
10	Van der Steen 2016 [40]	The psychological impact of prenatal diagnosis and disclosure of susceptibility loci: First impressions of parents' experiences
11	Vears 2016 [39]	Parents' experiences with requesting carrier testing for their unaffected children
12	Wilkins 2016 [38]	"It wasn't a disaster or anything": Parents' experiences of their child's uncertain chromosomal microarray result
13	Malek 2017 [37]	Parental Perspectives on Whole-Exome Sequencing in Pediatric Cancer: A Typology of Perceived Utility
14	Palomaki 2017 [36]	The clinical utility of DNA-based screening for fetal aneuploidy by primary obstetrical care providers in the general pregnancy population
15	Stivers 2017 [35]	The actionability of exome sequencing testing results
16	Barton 2018 [34]	Pathways from autism spectrum disorder diagnosis to genetic testing
17	Desai 2018 [33]	Impacts of variants of uncertain significance on parental perceptions of children after prenatal chromosome microarray testing
18	Harrington 2018 [32]	Parental perception and participation in genetic testing among children with Autism Spectrum Disorders
19	Szczepura 2018 [31]	UK families with children with rare chromosome disorders: Changing experiences of diagnosis and counselling
20	Williams 2018 [47]	Impact of a patient-facing enhanced genomic results report to improve understanding, engagement, and communication
21	Wou 2018 [52]	Parental perceptions of prenatal whole exome sequencing
22	Wynn 2018 [53]	Diagnostic exome sequencing in children: A survey of parental understanding, experience and psychological impact
23	Inglese 2019 [50]	New developmental syndromes: Understanding the family experience
24	Malek 2019 [49]	Responsibility, culpability, and parental views on genomic testing for seriously ill children

25	Taber 2019 [48]	Clinical utility of expanded carrier screening: results-guided actionability and outcomes
26	Aldridge 2020 [53]	Rapid genome-wide sequencing in a neonatal intensive care unit: A retrospective qualitative exploration of parental experiences
27	Berrios 2020 [54]	Parents of newborns in the NICU enrolled in genome sequencing research: hopeful, but not naïve
28	Brett 2020 [55]	Parental experiences of ultrarapid genomic testing for their critically unwell infants and children
29	Cakici 2020 [56]	A Prospective Study of Parental Perceptions of Rapid Whole-Genome and -Exome Sequencing among Seriously Ill Infants
30	Luksic 2020 [57]	A qualitative study of Latinx parents' experiences of clinical exome sequencing
31	Mollison 2020 [58]	Parents’ perceptions of personal utility of exome sequencing results
32	Riggan 2020 [59]	Family experiences and attitudes about receiving the diagnosis of sex chromosome aneuploidy in a child
33	Sandow 2020 [60]	Parental experiences and genetic counsellor roles in Pierre Robin sequence
